# Supplementary material for: Chromothripsis during telomere crisis is independent of NHEJ, and consistent with a replicative origin
Source: Genome Res. 2019 May;29(5):737–49. doi: 10.1101/gr.240705.118 (PMC6499312; doi:10.1101/gr.240705.118)
Supplement: Supplemental Material [file supp_gr.240705.118_Supplemental_file_1.zip › contigs/annotated_contigs/DB55/contig.3.DB55_length_293_mean_cov_11.7781569966.docx]

**DB55_length_293_mean_cov_11.7781569966**

```
TCAGCCAGGAGACAGAAACTTAAGATCAAAAAGGAGTTTCTGAAAGGCCTGCCTGAATGTATTAATCGATATTATTTGATGTCTACAAA
 >chr7:154586521-154586643 - E=4e-62
ACCCGCCACAAGGTGACCTGCTTCTGGGGGCCC|TGATGGAGCAGCG|TGGGGTGGGGGTGGGGGTGGGGGTGGGGGTGG|GG|GGGCC
                                               >chr6:42930963-42930997 + E=4e-09 p=1e
CATCCCATGGGGCGAGGGTGAGGCCGTGGGCCGCCTGCCTGCTCCACCCGGGCAACCTCTCTTACACTTCATTTTTCCTTTGCTCACTC
-02 >chr7:154586403-154586529 - E=1e-64
TTTGAGCATTTTAATTTTCTTTCCCCTACG
```
